# Large-Strain Hyperelastic Constitutive Model of Envelope Material under Biaxial Tension with Different Stress Ratios

**DOI:** 10.3390/ma11091780

**Published:** 2018-09-19

**Authors:** Zhipeng Qu, Wei He, Mingyun Lv, Houdi Xiao

**Affiliations:** 1 School of Aeronautic Science and Engineering, Beihang University, Beijing 100191, China; lv503@buaa.edu.cn (M.L.); xhdbuaa@126.com (H.X.); 2 AVIC Special Vehicle Research Institute, Jingmen 448035, China; sleet002@163.com; 3 College of Aerospace Engineering, Nanjing University of Aeronautics and Astronautics, Nanjing 210016, China

**Keywords:** envelope material, biaxial tension, constitutive model, stress ratio

## Abstract

This paper reports the biaxial tensile mechanical properties of the envelope material through experimental and constitutive models. First, the biaxial tensile failure tests of the envelope material with different stress ratio in warp and weft directions are carried out. Then, based on fiber-reinforced continuum mechanics theory, an anisotropic hyperelastic constitutive model on envelope material with different stress ratio is developed. A strain energy function that characterizes the anisotropic behavior of the envelope material is decomposed into three parts: fiber, matrix and fiber–fiber interaction. The fiber–matrix interaction is eliminated in this model. A new simple model for fiber–fiber interaction with different stress ratio is developed. Finally, the results show that the constitutive model has a good agreement with the experiment results. The results can be used to provide a reference for structural design of envelope material.

## 1. Introduction

The stress of stratospheric airship envelope material usually comes from two or more directions in practical application; thus, it is necessary to test envelope material by biaxial or multiaxial tension. In this paper, the mechanical properties of envelope materials are studied by biaxial tensile testing. There are mainly three kinds of biaxial tensile tests [1]: (1) the bursting test; (2) the cylinder test; and (3) the plane biaxial test. In the bursting test, the ration of warp and weft stress is fixed. In the cylinder tests, the effect of joints on the results should be considered. In plane biaxial tensile tests, there are two main types [2]: (1) tests using a single loading system and (2) tests using two or more independent loading systems. The stress ratio of warp and weft is limited when adopting the first loading. Taking secondary kinds of loading forms can achieve different stress ratios of warp and weft. In this paper, the mechanical properties of envelope material under different loading ratios is analyzed using secondary loading forms.

Biaxial tension can reflect the true envelope material’s tensile properties regarding tensile force under in-plane biaxial tensile tests; as such, it has been widely used. Many scholars have contributed a great deal to biaxial tensile tests. Lecompte et al. [3] proposed an inverse method to determine the engineering constants of a glass-fiber-reinforced epoxy though a biaxial tensile test. Makris et al. [4] optimized a biaxial tensile specimen using a sequential quadratic programming optimization method, so that the stress in the central region of the sample was uniform and the failure occurred in the central region. Kawabata et al. [5] studied the theory of uniaxial tensile and biaxial tensile properties in a general form of plain-weave fabrics. Moreno et al. [6] studied the failure envelope under biaxial tensile loading for chopped glass-reinforced polyester composites. Galliot et al. [7] proposed a simple model, describing the non-linear biaxial tensile behavior of Polyvinyl chloride (PVC)-coated polyester fabrics. Dinh et al. [8] proposed a new elastic material for coated fabric, based on uniaxial and biaxial tensile test data. Chen et al. [9] studied the tensile properties of the envelope fabric, Uretek-3216A, based on mono-uniaxial, uniaxial cyclic loading, and biaxial cyclic loading. Chen et al. [10] study the mechanical behaviors of laminated fabric Uretek-3216LV by uniaxial and biaxial tensile tests. Shi et al. [11] study the biaxial strength of three woven fabric composite materials under biaxial tensile experiments.

Many research works have been in progress for the constitutive modeling of envelope material. Xue et al. [12] studied a non-orthogonal constitutive model for characterizing woven composites under large deformations. However, their study only considered the constitutive model of fiber fabric without considering the mechanical properties of the matrix. Nayfeh et al. [13] developed a nonlinear constitutive relationship for plain textile, which was based on micromechanical behavior. Guo et al. [14] studied the mechanical behavior of an incompressible neo-Hookean material, directionally reinforced with a generalized neo-Hookean fiber. Li et al. [15] developed a constitutive thermoviscoelastic model for thin films that was based on the free volume theory, and the model was experimentally validated. Milani et al. [16] developed a new constitutive model relating to the behavior of fiber, matrix, and the interaction between both, to represent the mechanical properties of a fiber–matrix composite material. Hosoi et al. [17] studied the fatigue life of carbon fiber reinforced plastic materials.

This paper aims to investigate the large strain behavior of envelope materials under biaxial tension. The structure of this paper is as follows: In Section 2, the design of flexible composite material and biaxial tension tests are introduced. In Section 3, a new constitutive model is established. In Section 4, the new constitutive model is validated and a detailed discussion of each term of the constitutive model is presented. In Section 5, a brief summary and the conclusions is provided. It is hoped that this research will provide a good understanding of the biaxial tensile failure of envelope materials, and will provide technical support for the structural design of stratosphere airships.

## 2. Materials and Methods

### 2.1. Material

**Figure 1:**
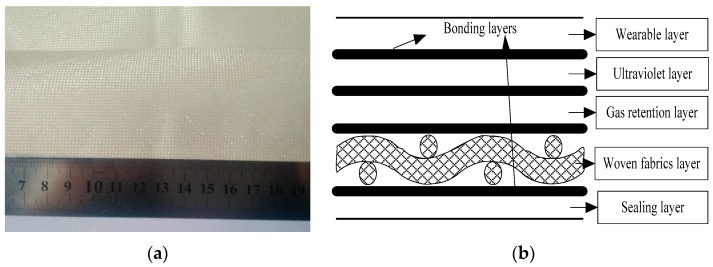
The envelope materials (**a**) macro morphology; (**b**) envelope layout.

Stratosphere airship envelope materials, a multi-layer flexible composite material, Uretek-3216LV (envelope material is provided by China Special Vehicle Research Institute of Hubei, Wuhan, China) [10], shown in Figure 1, with a thickness of 0.21 mm, and a surface density of 200 g/m^2^ was used. The stratospheric airship envelope material was composed of five functional layers. Functional layers were composed of a wearable layer, ultraviolet layer, gas retention layer, sealing layer, and a woven fabrics layer.

### 2.2. Biaxial Tensile Tests

**Figure 2:**
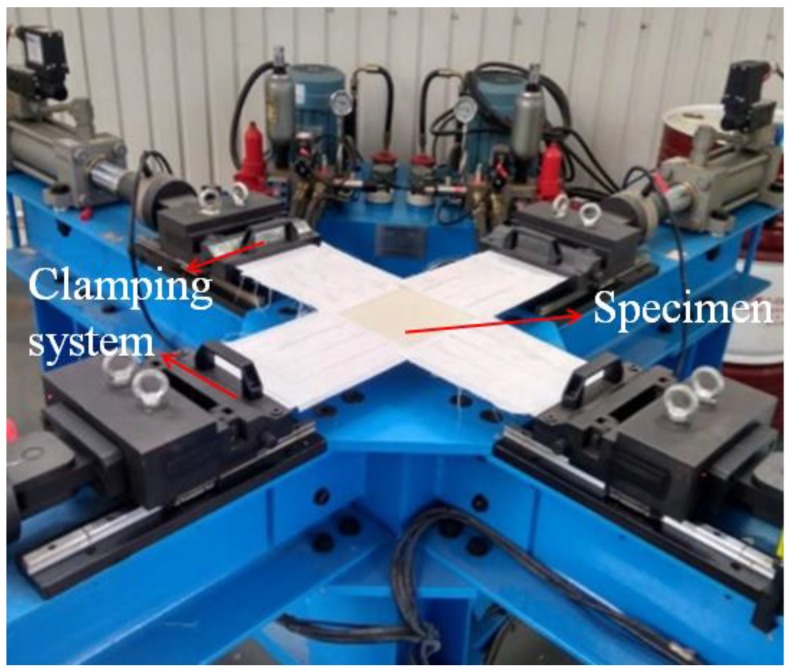
Biaxial test machine.

The biaxial test machine used in the experiments is shown in Figure 2. The biaxial tensile testing system mainly consisted of three relatively independent subsystems: (1) a rack system; (2) hydraulic system; and (3) control system. The maximum tensile load of the biaxial tensile testing machine was 20 kN. A film biaxial tension machine (the biaxial tensile machine is provided by Academy of Opto-electronics, Chinese Academy of Sciences of Beijing, Beijing, China) can achieve a variety of different tensile ratios of loading. The ambient temperature was 25 ± 3 °C, and the relative humidity of the laboratory was 50%. The different loading ratios are shown in Table 1.

### 2.3. Dimension of Cruciform Specimen

**Figure 3:**
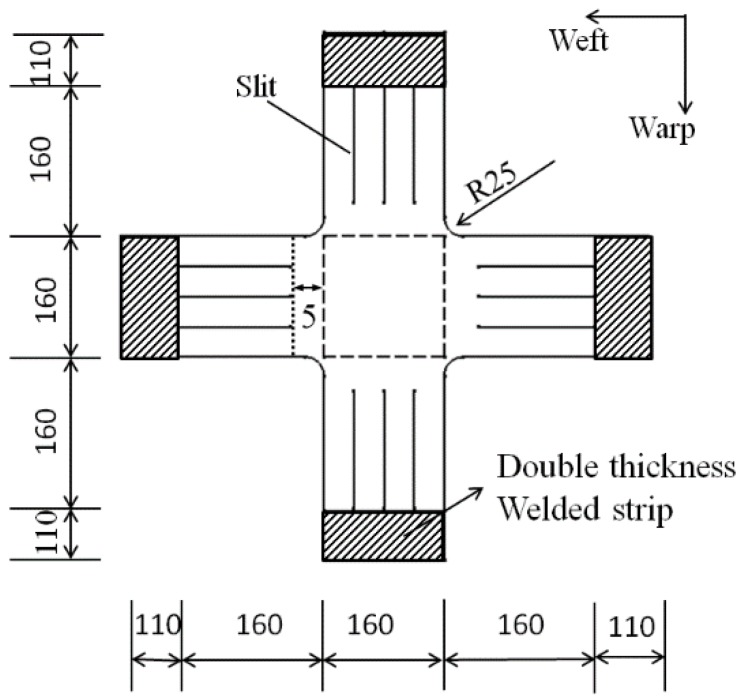
Dimensions of the specimen for biaxial tensile tests (unit in mm).

The dimensions of the specimen for the biaxial tensile tests are shown in Figure 3. The cross area of the specimen (dashed box in Figure 3) was 160 mm^2^ × 160 mm^2^ and the shape of the cross corner was rounded with a radius of 25 mm. The effective length of the arm was 160 mm. The double welded zone was the clamping area and was 110 mm in length. The end of the clamping region had a ring with a radius of 20 mm.

### 2.4. Result of Biaxial Tensile Test

**Figure 4:**
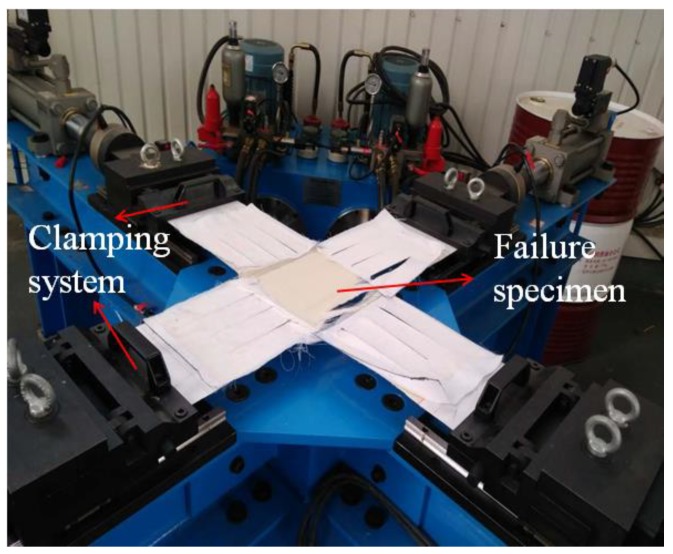
Failure mode of the biaxial tensile specimen.

Biaxial tensile tests of the envelope material were carried out. The loading rate of the biaxial stretching was 8 kN/(m·min) under an equal loading rate of warp and weft. The failure mode of the biaxial tensile specimen is shown in Figure 4. The failure of the biaxial tensile specimens occurred in the central region. The central region of the specimen was stretched and broken. Fracture of the center region of the specimen, without shrinkage deformation, and the fiber yarn bundle fracture were almost at the same time; the fiber yarn bundle and matrix functional layers occurred only in the local area around the slit, with the phenomenon of the fiber yarn bundle pull-out and fracture segment.

**Figure 5:**
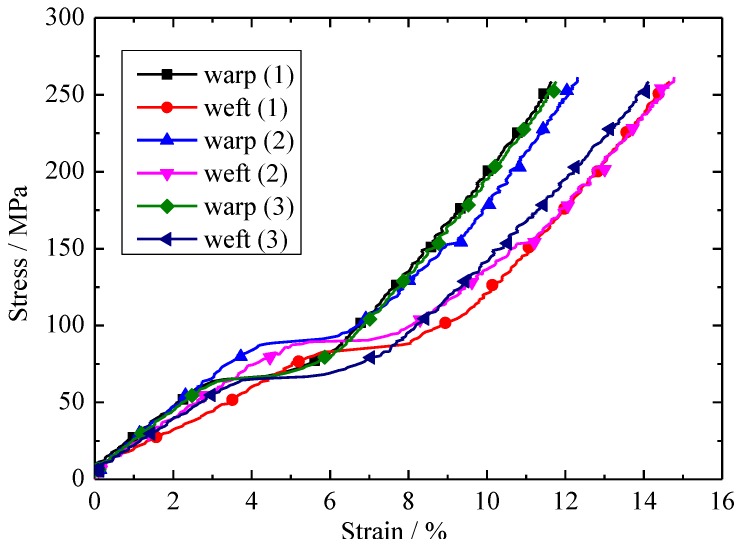
Stress–strain curves of the biaxial tensile tests under equal loading ratio in warp and weft directions.

**Figure 6:**
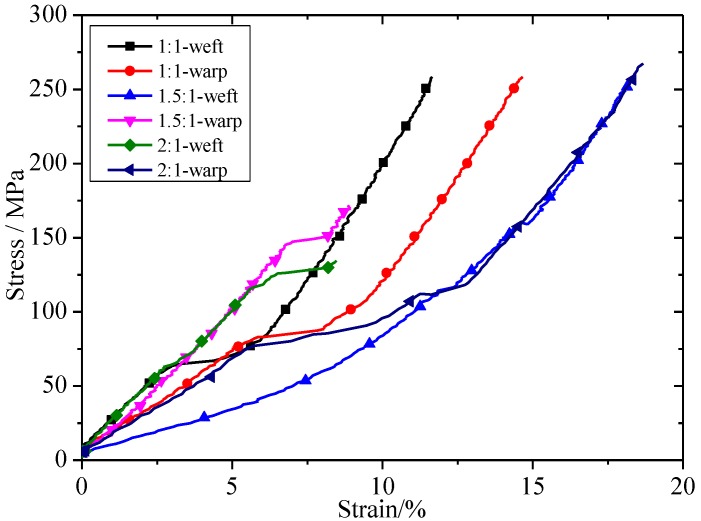
Stress–strain curves of the biaxial tensile tests under different loading ratios in warp and weft directions.

The stress–strain curves of the biaxial tensile tests under equal stress ratios of warp and weft are shown in Figure 5 and Figure 6. The preload was 200 N. Warp (1) represents the warp of the first biaxial tensile test. As can be seen in Figure 5, the stress-strain curves of the three specimens are similar, the failure strength and failure strain of the three groups had little difference (10%). the possible reason is due to the discrete type of the envelope material.

The stress–strain curves of biaxial tensile test under different stress ratios of warp and weft are shown in Figure 6. In the 1.5:1-weft, 1.5:1 represented the stress ratio and weft represented the tensile direction. It can be seen in Figure 6 that the stress–strain curves of the envelope material, with different stress ratios, had the same trend. The difference was that the displacement elongation varied slightly under different stress ratios. This may be due to the discrete type of envelope material.

## 3. A New Constitutive Model for Envelope Materials under Large Strains

Envelope materials experience large deformations when subjected to biaxial loading. The constitutive model is difficult to obtain, owing to the geometric nonlinearity and material nonlinearity of an envelope material. In this section, based on fiber-reinforced continuum mechanics, a new constitutive model for envelope materials under different loading ratios of warp and weft was developed.

To simplify the analytical model, several assumptions were made, as follows:

1. Bonding between the substrate fabric and any other functional layers was assumed to be perfect.
2. The envelope material was made up of matrix and fabric components. In addition to the fabric layer, other functional layers are regarded as the matrix.
3. The shear stress mainly contributed to the matrix stress. In this work, the modulus of shear was kept constant.
4. The failure mode of the envelope material during biaxial tension was a brittle fracture. The failure of the matrix and fiber stress occurred at the same time.

### 3.1. Constitutive Model Theory with Equal Stress Ratios

Spencer et al. [18] adopted a hyperelastic model, which used a particular Helmholtz free energy function to predict the mechanics of solids. It is assumed that the Helmholtz free energy function is a scalar function of the right Cauchy–Green deformation tensor, C = F^T^F, and fiber directional vector a_0_. Here, F is the deformation gradient tensor. The large strain response of the envelope was assumed to originate from the resistance of the matrix, fibers, fiber interactions and fiber–matrix interaction. Therefore, the strain energy can be divided into four parts, i.e.:

(1) $$W=W(C,a_{0})=W^{M}+W^{F}+W^{FF}+W^{FM}$$

where *W^M^* is the strain energy contribution from matrix resistance, *W^F^* is the strain energy contribution from the fiber stretch, *W^FF^* is the strain energy contribution from the fiber–fiber interaction due to the weaving, and *W^FM^* is the strain contribution from the fiber-matrix shear interaction.

According to the invariant theory, Equation (1) can be rewritten as follows:

(2) $$W({C,a}_{0})=W(I_{1},I_{2},I_{3},I_{4a},I_{5a})$$

According to the literature [18], free energy may be a function of the following invariants:

(3) $$I_{1}(C)=\mathrm{tr}(C)=C:I$$

(4) $$I_{2}=\frac{1}{2}[\mathrm{tr}{\left(C\right)}^{2}-\mathrm{tr}(C^{2})]$$

(5) $$I_{3}(C)=\det(C)=J^{2}$$

(6) $$I_{4a}(C,a_{0a})=a_{0a}\cdot Ca_{0a}$$

(7) $$I_{5a}(C,a_{0a})=a_{0a}\cdot C^{2}a_{0a}$$

where *I*_1_, *I*_2_, *I*_3_ are invariants of the strain tensor and *I*_4a_ is the squares of the stretches along the fiber directions [19]. *I*_5a_ is the fourth power of the stretching along the fiber directions.

In continuum mechanics, under isothermal conditions, the second law of thermodynamics can be represented as the Clausius–Duhem inequality. For perfectly-elastic materials, and if thermal effects are ignored (purely mechanical theory), the inequality degenerates into the following equality:

(8) $$\Omega =S:\dot{C}/2-\dot{W}=(S-2\frac{\partial W}{\partial C}):\dot{C}/2=0$$

where Ω is the entropy generation and S is the second Piola–Kirchhoff stress tensor. Dot (·) represents the material time derivative. The second Piola–Kirchhoff stress tensor components can be derived from the free energy, as follows:

(9) $$S=2\frac{\partial W}{\partial C}=2\frac{\partial W(I_{i})}{\partial I_{i}}\frac{\partial I_{i}}{\partial C}$$

where summation over dummy index i is implied. Once the shape of Ψ is known, the second Piola–Kirchhoff stress can be derived.

In the literature [20], a classic neo-Hookean model is used to represent matrix response *W^M^*:

(10) $$W^{M}{=c}_{1}/2(\bar{I_{1}}-3)$$

where ${\bar{I}}_{1}$ is the first deviatoric invariant:

(11) $${\bar{I}}_{1}=J^{-2/3}I_{1}$$

The fibers’ contribution to the extension is captured with an exponential function; the expression is as follows [16]:

(12) $$W^{F}=\frac{k_{1a}}{{2k}_{2a}}\sum_{a=1}^{2}{\{\exp[k}_{2a}{\left(I_{4a}-1\right)}^{2}]-1\}$$

In this paper, label a = 1, 2 denotes the warp and weft direction, respectively.

The fiber–fiber strain energy contribution [21] is expressed as follows:

(13) $$W^{FF}=k(I_{41}-1)(I_{42}-1)$$

The fiber–matrix strain energy contribution [22] is expressed as follows:

(14) $$W^{FM}=f(I_{4})[\frac{I_{4}}{I_{3}}(I_{5}-I_{1}I_{4}+I_{2})-1]$$

It is easy to prove *W^FM^* equal zero when biaxial tensile tests in warp and weft directions for envelope material.

In summary, the strain energy can be expressed as follows when the envelope material is under biaxial tension along the warp and weft directions:

(15) $$W=W(C,a_{0})=W^{M}+W^{F}+W^{FF}$$

After extensive simplification, the second Piola–Kirchoff stress tensor can be expressed as follows:

(16) $$\begin{array}{l} S=-{\mathrm{pC}}^{-1}+c_{1}I+[2k_{11}(I_{41}-1)\exp(k_{21}{\left(I_{42}-1\right)}^{2})+2k(I_{42}-1)]A_{01}\\ \;+[2k_{12}(I_{42}-1)\exp(k_{22}{\left(I_{42}-1\right)}^{2})+2k(I_{41}-1)]A_{02} \end{array}$$

Here, p is the hydrostatic pressure and A_0*i*_ is defined as follows:

(17) $$A_{0i}=a_{0i}⊗a_{0i}$$

The relationship between the second Piola–Kirchoff stress tensor and the Cauchy stress tensor σ is as follows:

(18) $$\sigma =J^{-1}F\cdot S\cdot F^{T}$$

The planar Cauchy stress components can be described as follows:

(19) $$\begin{array}{l} \sigma =-pI+c_{1}C^{T}+[2k_{11}(I_{41}-1)\exp(k_{21}{\left(I_{41}-1\right)}^{2})+2k(I_{42}-1)]A_{1}\\ \;+[2k_{12}(I_{42}-1)\exp(k_{22}{\left(I_{42}-1\right)}^{2})+2k(I_{41}-1)]A_{2} \end{array}$$

Here, *A_i_* is defined as follows:

(20) $$A_{i}=Fa_{0i}⊗Fa_{0i}$$

The warp and weft Cauchy stress components can be described, respectively, as follows:

(21) $$\sigma_{11}=c_{1}(\lambda_{1}^{2}-\lambda_{1}^{-2}\lambda_{2}^{-2})+2k_{11}\lambda_{1}^{2}(\lambda_{1}^{2}-1)\exp(k_{21}{\left(\lambda_{1}^{2}-1\right)}^{2})+2k(\lambda_{2}^{2}-1)$$

(22) $$\sigma_{22}=c_{1}(\lambda_{2}^{2}-\lambda_{1}^{-2}\lambda_{2}^{-2})+2k_{12}\lambda_{2}^{2}(\lambda_{2}^{2}-1)\exp(k_{22}{\left(\lambda_{2}^{2}-1\right)}^{2})+2k(\lambda_{1}^{2}-1)$$

Here, λ is the principal stretch.

### 3.2. Constitutive Model Theory with Different Stress Ratios

The purpose of the present study is to investigate the large strain response of envelope materials under different loading ratios. The strain energy can be divided into three parts when an envelope material is under the biaxial tension of warp and weft: Matrix energy, fiber energy, and fiber–fiber energy. In this section, the matrix energy parameter and the fiber energy parameters are assumed to be constant. The fiber–fiber energy is related to the stress ratio. It is assumed that the stress ratio between the warp and weft directions is σ_1_:σ_2_ = C (C ≥ 1). In this section, the warp loading speed is constant. The weft fiber–fiber strain energy is assumed to be as follows:

(23) $$W^{FF}=k_{C}(I_{41}-1)(I_{42}-1)$$

The weft Cauchy stress can be described as:

(24) $$\begin{array}{l} \sigma_{22}=c_{1}(\lambda_{2}^{2}-\lambda_{1}^{-2}\lambda_{2}^{-2})+2k_{11}(\lambda_{2}^{2}-1)\exp(k_{21}{\left(\lambda_{2}^{2}-1\right)}^{2})+2k_{C}(\lambda_{1}^{2}-1)\\ C\geq 1,k_{C}=k/C\\ C<1,k_{C}=k\cdot C \end{array}$$

Then, the warp Cauchy stress can be determined through the stress ratio between the warp and weft directions, i.e.:

(25) $$\sigma_{11}=C\sigma_{22}=C(c_{1}(\lambda_{2}^{2}-\lambda_{1}^{-2}\lambda_{2}^{-2})+2k_{11}(\lambda_{2}^{2}-1)\exp(k_{21}{\left(\lambda_{2}^{2}-1\right)}^{2})+2k_{C}(\lambda_{1}^{2}-1))$$

### 3.3. Identification of the New Constitutive Model Parameters

First of all, the identification of new constitutive model parameters was introduced. In order to obtain the coefficients of Equations (24) and (25), a residual objective function was introduced. The residual objective function is given by:

(26) $$\Phi (\sigma )={\sum_{i=1}^{N}\left(\sigma_{11,i}^{c}-\sigma_{11,i}^{\exp}\right)}^{2}+{\sum_{i=1}^{N}\left(\sigma_{22,i}^{c}-\sigma_{22,i}^{\exp}\right)}^{2}$$

where *c* represents the calculated value, exp represents the experimental value, and *N* represents the number of experimental points.

The new constitutive model had six parameters. The specific solution procedure was as follows:

(1) k_11_ (warp fiber initial stiffness) and k_12_ (weft fiber initial stiffness) represent the initial fiber stiffness. The two parameters can be obtained by the initial slope of the biaxial tensile tests.
(2) c_1_ (matrix shear modulus) and k (fiber-fiber interaction parameter in equal stress ratio) can be solved by the small strain in the biaxial tensile tests. The two parameters can be obtained by the following equation [21]:
  (27) $$\begin{array}{l} {\frac{\partial \sigma_{11}}{\partial \lambda_{1}}|}_{\begin{array}{l} \lambda_{1}=1\\ \lambda_{2}=1 \end{array}}=4c+4k_{11}\\ {\frac{\partial \sigma_{22}}{\partial \lambda_{1}}|}_{\begin{array}{l} \lambda_{1}=1\\ \lambda_{2}=1 \end{array}}=2c+4k\\ {\frac{\partial \sigma_{11}}{\partial \lambda_{2}}|}_{\begin{array}{l} \lambda_{1}=1\\ \lambda_{2}=1 \end{array}}=2c+4k\\ {\frac{\partial \sigma_{22}}{\partial \lambda_{2}}|}_{\begin{array}{l} \lambda_{1}=1\\ \lambda_{2}=1 \end{array}}=4c+4k_{12} \end{array}$$
(3) Parameters k_21_ (warp fiber large strain control parameter) and k_22_ (weft fiber large strain control parameter) are related to the slope of stress–strain curves under large strains. The two parameters can be obtained by biaxial tensile tests with large strain slopes.

### 3.4. Validation of New Constitutive Model

The new constitutive model was validated against experimental data for coated fabrics [23]. The identification of the new constitutive model was employed as an unconstrained least square method. The Levenberg–Marquardt solution algorithm was employed to solve the nonlinear least squares problems. The constitutive model parameters can be derived from Equations (24) and (25). The parameters of the new constitutive model for coated fabrics under various stress ratios are shown in Table 2.

**Figure 7:**
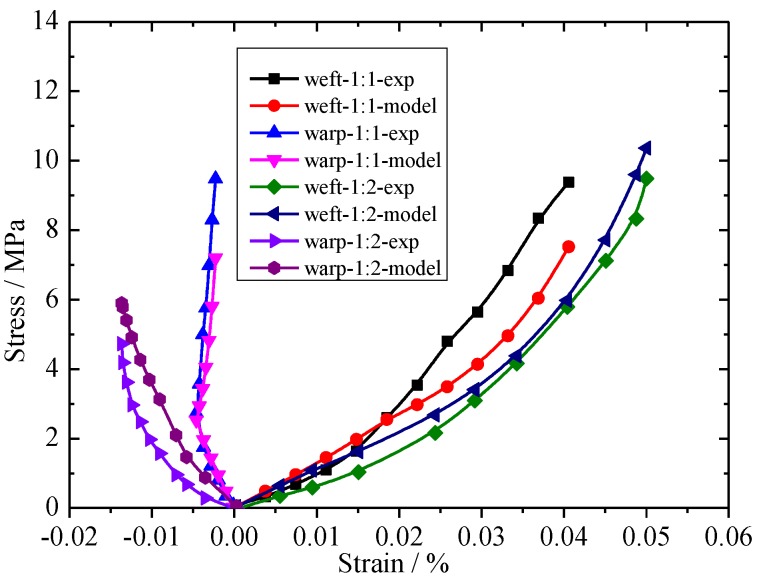
Comparison between the experimental [23] and predicted models for coated fabrics.

A comparison of the test results and the predicted model values is shown in Figure 7. In weft-1:1-exp, weft represents the biaxial tensile in the weft direction, 1:1 represents the stress ratio, and exp represents the experimental results. Good agreement between the experimental and predicted models was observed for the coated fabrics. The constitutive model of envelope materials is mainly used for biaxial tensile tests with large strains; thus, the error is large when the biaxial tensile test has a small strain.

## 4. Results and Discussion

The new constitutive model provides the possibility of predicting the mechanical responses of envelope material Uretek-3216LV. First of all, the constitutive parameters of the envelope materials in the biaxial tensile tests, with equal stress ratios, were obtained. The parameters of the model for the envelope material under equal loading ratios are shown in Table 3.

**Figure 8:**
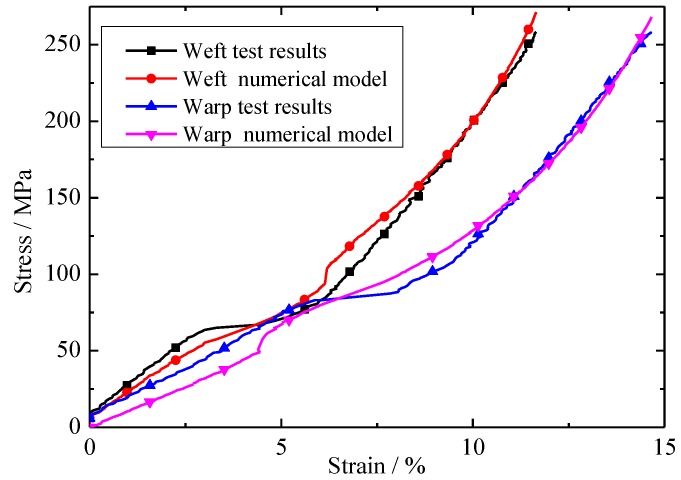
Comparison of test results and numerical models with a stress ratio of 1:1 (warp:weft).

A comparison of the biaxial tensile tests and the numerical results under equal loading ratios is shown in Figure 8. It can be seen from the figure that the new constitutive model shows good agreement with the corresponding experimental curves. The new constitutive model can capture the three stages of the stress–strain curves. The value of R^2^ between the experimental data and the new constitutive model was 0.98. Therefore, the new constitutive model is an acceptable model in predicting envelope material stress–strain behavior.

**Figure 9:**
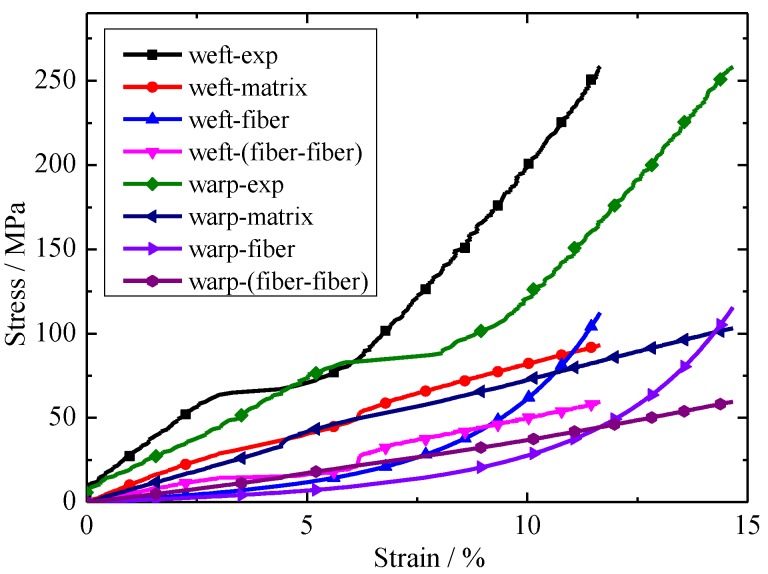
Relationship between the each constituent of stress and experimental stress with displacement under a stress ratio of 1:1 (warp:weft).

The relationship between each part of the stress and the experiment stress with respect to strain is shown in Figure 9. Here weft-exp represents the experimental value of envelope material under biaxial tension in the weft direction, weft-fiber represents the stress of the fibers under biaxial tension in the weft direction. Other nomenclature rules such as weft-exp are similar meaning to warp-exp. It can be seen from Figure 9 that the contribution of each part of the stress contribution to the experimental stress is different. Fiber stress and matrix stress are of great importance for new constitutive models, the contribution of fiber–fiber interactions is somewhat smaller. The contribution of each part to the model in the warp tensile direction has little impact in the warp tensile direction. The difference is that, under the same stress conditions, the elongation is different.

The predictive values of the envelope material under different ratios are shown in Table 4. The test results have a good agreement with the predictive values. The new constitutive model is an acceptable model in predicting envelope material stress–strain behavior.

**Figure 10:**
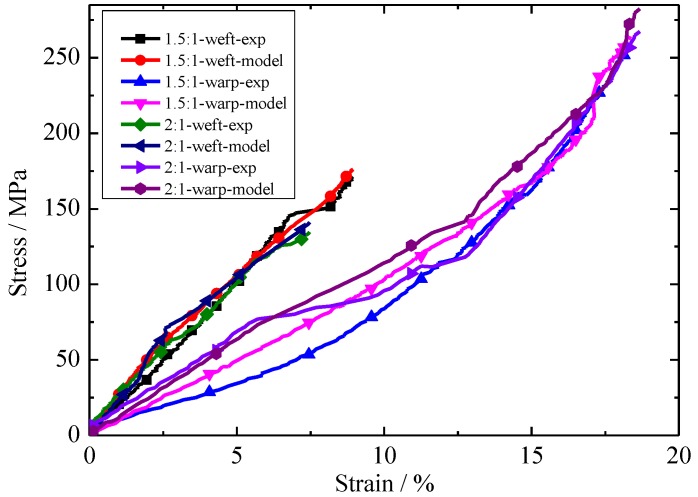
Comparison of test results and numerical models under different stress ratios.

A comparison of biaxial tensile tests and the numerical results under different loading ratios is shown in Figure 10. In 1.5:1-weft-exp, 1.5:1 represents the loading ratio on the envelope material, weft represents the loading direction, and exp represents the experimental results. The rest of the symbols are similar to those of 1.5:1-weft-exp. The new constitutive model has a good agreement with the experimental results. The new constitutive model is an acceptable model in predicting envelope material stress–strain behavior with different stress ratios.

**Figure 11:**
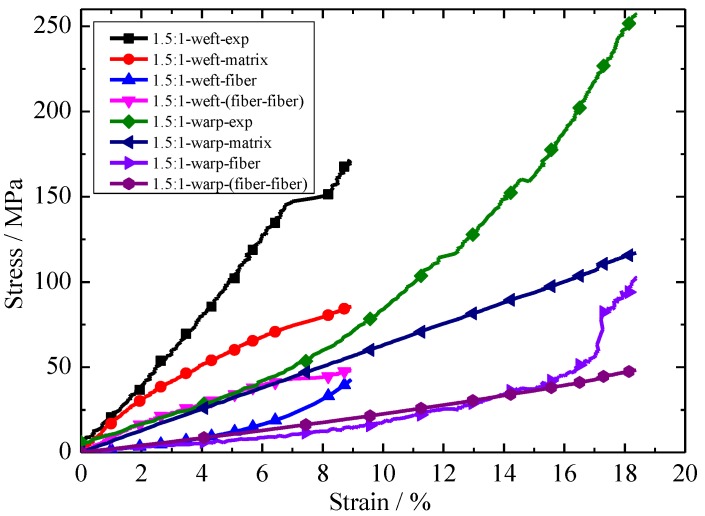
Relationship between each constituent of stress and experimental stress with strain under a stress ratio of 1.5:1 (warp:weft).

**Figure 12:**
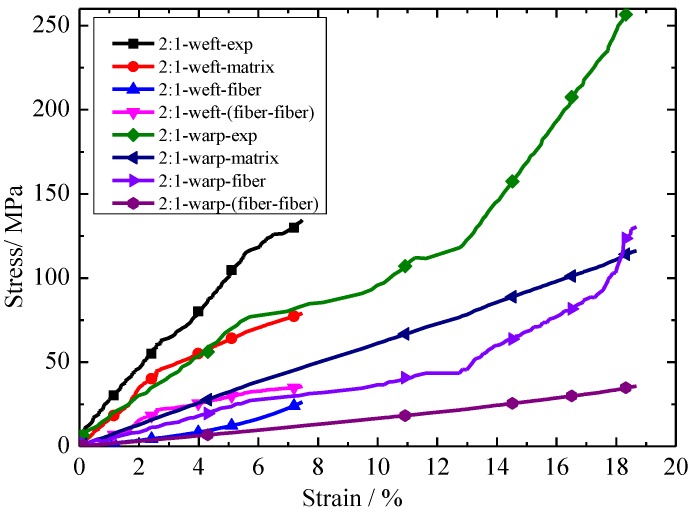
Relationship between each constituent of stress and experimental stress with strain under a stress ratio of 2:1 (warp:weft).

The relationships between each constituent of stress and experiment stress with strain under different stress ratios are shown in Figure 11 and Figure 12. Here, in 1.5:1-weft-exp, 1.5:1 represents the stress ratios, weft represents the biaxial tension in the weft direction, and exp represents the experimental results. Other nomenclature rules such as 1.5:1-warp-exp are similar meaning to 1.5:1-weft-exp. It can be seen from Figure 11 and Figure 12 that the contribution of each constituent of stress contribution to experimental stress is different. The contribution of stress of each part to experimental stress is: matrix > fiber > fiber-fiber. The contributions of matrix stress and fiber stress to experimental stress remained constant as the stress ratio increased. However, the interaction between fiber and fiber decreases with the increase in stress ratio.

## 5. Conclusions

An anisotropic hyperelastic constitutive model with different stress ratios was developed based on the mechanics of fiber-reinforced continuum mechanics. The strain energy function that characterizes the anisotropic behavior of the envelope material is divided into three parts: Fiber, matrix, and fiber–fiber interaction. The interaction stress of fiber and fiber is inversely proportional to the stress ratio. The results show that the new constitutive model has a good agreement with the experimental results.

The contribution of each constituent stress to the experimental results of the envelope material using different ratios was analyzed. The results showed that the contribution of matrix stress, fiber stress and interaction stress between fiber and fiber on the experimental stress gradually decreased. The contribution of matrix stress and fiber stress to experimental stress remained constant as the stress ratio increased, and the interaction between fiber and fiber decreased with the increase in stress ratio.

In the new constitutive model, the interaction between the fiber and matrix can be ignored. This research has shown that the fiber–fiber interaction has a great influence on the constitutive relationship under biaxial tension. To verify this result, future work will study the effect of a fiber–fiber on biaxial tension for other kinds of lamianted fabrics.

## Figures and Tables

**Table 1:** Layout of the experimental plan.

Loading Ratios	Warp (N·mm^−1^/min)	Weft (N·mm^−1^/min)	Number of Test Specimens
1:1	40	40	3
1.5:1	40	27	3
2:1	40	20	3

**Table 2:** The parameters of the model for envelope materials under equal loading ratios.

c_1_	k_11_	k_21_	k_12_	k_22_	k	k_c_ (c = 0.5)
(MPa)	(Mpa)	(1)	(Mpa)	(1)	(Mpa)	(1)
11	32	10	28	−20	36	7

k_c_ = fiber-fiber interaction parameter in different stress ratios.

**Table 3:** The parameters of the model for envelope material under equal loading ratios.

Loading Ratio	c_1_	k_11_	k_21_	k_12_	k_22_	k	R^2^
	(MPa)	(MPa)	(1)	(MPa)	(1)	(MPa)	(1)
1:1	146.48 ± 18.62	25.57 ± 4.86	17.14 ± 1.23	40.53 ± 12.52	24.78 ± 2.32	76.00 ± 9.71	0.98

**Table 4:** Comparison of test results and predictive results under different ratios.

Loading Ratios	k_c_ (MPa)	
	Tests	Predicted
1.5:1	47.77 ± 4.86	50.72 ± 6.94
2:1	37.86 ± 2.98	38.05 ± 4.03
